# A phase I/II study of gemcitabine and fractionated cisplatin in an outpatient setting using a 21-day schedule in patients with advanced and metastatic bladder cancer

**DOI:** 10.1038/sj.bjc.6602112

**Published:** 2004-08-03

**Authors:** S A Hussain, D D Stocken, P Riley, D H Palmer, D R Peake, J I Geh, D Spooner, N D James

**Affiliations:** 1 Cancer Research UK Institute for Cancer Studies, University of Birmingham B15 2TT, UK; 2 Queen Elizabeth Hospital, Edgbaston, Birmingham B15 2TH, UK

**Keywords:** bladder cancer, gemcitabine, fractionated cisplatin, outpatient, chemotherapy

## Abstract

A randomised phase III trial of MVAC (methotrexate, vincristine, doxorubicin, cisplatin) *vs* gemcitabine and cisplatin (GC) (G 1000 mg m^−2^ days 1, 8, and 15 plus C 70 mg m^−2^ day 2, q 4 wks) indicated GC had similar efficacy and lower toxicity (*JCO* 2000). Significant haematologic toxicities in the GC arm occurred on day 15, necessitating dose adjustments in 37% of cycles. We conducted a phase I/II dose escalation trial using GC on a 21-day cycle, with G and C split between days 1 and 8. The objective of the study to define maximum-tolerated dose and dose-limiting toxicity (DLT), objective response rate, and overall survival. In all, 32 patients with locally advanced, relapsed, or metastatic disease received: dose level 1, G/C 1000/35; level 2, 1100/35; level 3, 1200/35; level 4, 1200/45 mg m^−2^ (G and C given on days 1 and 8 every 3 wks). A total of 19 patients had glomerular filtration rate <60 ml min^−1^ and 19 patients had metastatic disease. Dose-limiting toxicity was haematologic (grade 4 thrombocytopenia) at dose level 2. Of 151 cycles, at day 15, platelets were <100 in 61 cycles; neutrophils <0.5, platelets <50 in 26 cycles. Only seven cycles were deferred due to haematological toxicity; four for renal toxicity (chemotherapy instituted posthydration). Overall response rate was 65.5% on an intention-to-treat analysis (75% [21/28] for assessable patients), with four complete responses (12.5%) and 17 partial responses (53%). After the median follow-up of 17.2 months (range 13.1–32.4 months), 12 patients remain alive. The overall median survival was 16 months (range 10.1–26.6 months). G plus C every 3 weeks is active and well tolerated in an outpatient setting, even in patients receiving prior platinum-based regimens and with poor renal reserve.

Phase I and II trials in the 1980s demonstrated that metastatic urothelial transitional cell cancers are chemosensitive. Currently, systemic combination chemotherapy is the only treatment that may result in long-term survival for some patients with advanced or metastatic disease. Although antitumour activity has been demonstrated with several agents, the median survival associated with single-agent therapy is 4–6 months. The median survival time for combination regimens (e.g. methotrexate+vinblastine, or doxorubicin+cisplatin) is approximately 8 months (Sternberg *et al*, 1989). It is known that pretreatment prognostic features have an impact on individual patient outcome, thus the variation in reported survival in patients treated with chemotherapy may be biased by these features (Bajorin *et al*, 1999; Calabro and Sternberg, 2002).

In 1985, the Memorial Sloan-Kettering cancer centre (MSKCC) investigated the four-drug regimen using MVAC (methotrexate, vincristine, doxorubicin, cisplatin) (Sternberg *et al*, 1985) in patients with TCC. They reported an overall response rate of 71%. These results were later confirmed in a series of 133 patients (Sternberg *et al*, 1989). Other studies using the MVAC regimen reported encouraging response rates including complete responses of 13–19% (Tannock *et al*, 1989; Igawa *et al*, 1990; Boutan-Laroze *et al*, 1991). Another study reported the use of MVAC in 21 patients with high-stage TCC. This study, with long-term follow-up, suggested that the durability of response was disappointing (Connor *et al*, 1989). Long-term follow-up evaluation of the intergroup trial confirmed that MVAC was superior to single-agent cisplatin in patients with advanced urothelial carcinoma. However, durable progression-free survival was rare (Saxman *et al*, 1997).

The combination of CMV (cisplatin, methotrexate, vincristine) has also been investigated with similar response rates to those reported with MVAC. A study of 58 patients with metastatic TCC reported complete responses (CR) in 14 of 50 evaluable patients and partial responses (PR) in 14 patients for an overall response rate of 56%. The median survival for CRs was 11 *vs* 7 months for PRs (*P*<0.05) and 6 months for nonresponders. Renal and haematological toxicities with this regimen were moderate (Harker *et al*, 1985).

Although response rates achieved using MVAC have been encouraging, this regimen has significant toxicity, particularly in older patients. This is relevant to bladder cancer since the median age of patients is approximately 68 years. Many of these patients have impaired renal function, which may compromise the safety of the use of cisplatin and increase the risk of toxicity. Indeed, a significant proportion of otherwise fit patients (around 50% in our institution) have a calculated glomerular filtration rate <60 mls min^−1^, the usual cutoff for combination studies with cisplatin. Treatment with MVAC can result in clinically significant myelosuppression (up to 25% incidence of neutropenic fever and a 3% drug-related mortality) and significant mucositis (up to 50% incidence of grade 2–3 mucositis) (Sternberg *et al*, 1985, 1989; Connor *et al*, 1989; Tannock *et al*, 1989; Igawa *et al*, 1990; Logothetis *et al*, 1990; Boutan-Laroze *et al*, 1991; Loehrer *et al*, 1992). Recent efforts to improve the outcome for patients with metastatic transitional cell carcinoma have focused on the identification of new drugs with single-agent activity and on their incorporation into platinum-based combination regimens. Paclitaxel, docetaxel, ifosfamide, and gemcitabine are among the most active new agents (Hussain and James, 2003).

A large phase III trial randomised 405 patients with stage IV TCC and with no prior chemotherapy exposure between GC (gemcitabine 1000 mg m^−2^ days 1, 8, and 15; cisplatin 70 mg m^−2^ day 2) or MVAC every 28 days for a maximum of six cycles. Overall survival, time to disease progression, time to treatment failure, and response rate (GC, 49%; MVAC, 46%) were similar in each arm. Owing to a higher incidence of neutropenic fever and mucositis, more hospital admissions were required for patients receiving MVAC (49 admissions for a total of 272 days) compared to the GC group (9 admissions for a total of 33 days), resulting in considerably greater hospital resource utilisation (Von Der Maase *et al*, 2000). Thus, although this trial was not designed to show equivalence of the two regimens, many have interpreted the results as showing therapeutic noninferiority and adopted the GC regimen as the new standard on the basis of better tolerability (De Wit *et al*, 2001).

## STUDY RATIONALE

Gemcitabine has shown activity in patients with bladder cancer both as a single-agent and in cisplatin-based combination regimens. Toxicities with this combination have been mild to moderate. The dose-limiting side effects have been mainly haematological, although grade 3 or 4 neutropenic fever is relatively infrequent. A large phase III trial comparing MVAC with GC showed that with the same efficacy, GC had a better toxicity profile (Von Der Maase *et al*, 2000). Most haematological toxicity of GC was encountered on day 15, necessitating dose modifications in 37% of cycles.

Based on these observations, we have investigated the use of GC utilising a 21-day schedule, omitting day 15 with the aim of reducing myelotoxicity, thus reducing treatment delays/dose modifications and increasing dose intensity. It was further hypothesised that splitting the cisplatin dose over days 1 and 8 may also reduce nephrotoxicity, extending the potential spectrum of patients eligible for treatment. This combination can be given in an outpatient setting, thus reducing hospital bed use and improving cost-effectiveness.

The study protocol was approved by the Local Research Ethics Committee.

### Study objectives

This was a combined phase I/II study of gemcitabine+cisplatin given on days 1 and 8 of a 21-day cycle in patients with locally advanced or metastatic transitional cell carcinoma of the bladder:

- *Primary objectives*: To establish the maximum-tolerated dose and dose-limiting toxicities.
- *Secondary Objectives*: To estimate the objective response rate using WHO criteria for response assessment. To estimate overall survival.

### Study treatments

Patients received up to a maximum of six cycles of treatment. All patients had blood count and clinical chemistry evaluation before each cycle of chemotherapy. Gemcitabine was given in 500 ml of 0.9% saline and infused over 30 min. This was followed by cisplatin given with adequate hydration using 0.9% saline pre- and post-treatment. All drugs were administered via a peripheral intravenous (i.v.) infusion line. Additional hydration, frusemide (i.v. or oral), potassium, and magnesium supplements were administered as necessary. Antiemetic therapy comprised 8 mg i.v. dexamethasone and 3 mg i.v. granisetron. The total duration of treatment was approximately 4 h and was carried out in the outpatient clinic.

### Study design

The aim of the phase I portion of this study was to define the MTD of this treatment schedule. A mixed escalation regimen was used as summarised in Table 1

**Table 1:** Planned dose escalation

**Dose level**	**Gemcitabine (mg m^−2^)**	**Cisplatin (mg m^−2^)**
1	1000	35
2	1100	35
3	1200	35
4	1200	45

.

Dose-limiting toxicity (DLT) was defined using NCI CTC criteria as follows: haematological toxicity, grade 4. Nonhaematological toxicity (except nausea/vomiting and alopecia), grade ⩾3. Starting at dose level 1, a minimum of three patients were to be recruited into each dose level. Once they had all completed one cycle of treatment, if no DLT was observed, subsequent patients were recruited to the next dose level. If one patient experienced DLT, the cohort was expanded to a total of six patients. If two or more patients experienced DLT, then this defined MTD at this dose level, otherwise dose escalation continued. Patients who were removed from the study for any reason other than toxicity and failed to complete the first cycle of treatment were to be replaced. The planned phase II dose was the dose level immediately below the MTD.

### Planned dose reductions for haematological toxicity

The myelosuppression seen with gemcitabine plus cisplatin (GC) tends to be short-lived and noncumulative, and so no account of nadir values was taken for subsequent cycles. All doses were administered according to the neutrophil and platelet counts in the 24-h period prior to treatment as summarised in Table 2

**Table 2:** Dose modifications for haematological toxicity

**ANC (× 10^9^ l^−1^), platelets (× 10^9^ l^−1^)**	**Gemcitabine**	**Cisplatin**
>1.5 and >100	100%	100%
0.5–1.5 or 50–100	75%	75%
<0.5 or <50	Delay^a^/omit^b^	Delay^a^/omit^b^

a Day 1 treatments delayed until haematologic status allows treatment. If delay is >3 weeks, the patient will be withdrawn from the study.

b Day 8 treatment is omitted.

ANC=absolute neutrophil count.

.

## PATIENTS AND METHODS

Patients with histologically confirmed muscle invasive primary transitional cell carcinoma of the bladder and with locally relapsed, locally advanced or metastatic disease were eligible for the study. No more than one prior systemic chemotherapy regimen for primary radical treatment at the time of disease presentation was permissible. Patients previously treated with radiotherapy were eligible 3 months post-treatment and after having recovered from any radiation-related toxicity.

The presence of one or more clinically or radiologically bidimensionally measurable lesion with one diameter of at least 2 cm was required.

Other criteria for patient entry into this trial were: age greater than 18 years; life expectancy ⩾12 weeks; WHO performance status 0–2; adequate haematological function (Hb ⩾10.0 g dl^−1^, neutrophils ⩾2.0 × 10^9^ l^−1^, platelets ⩾100 × 10^9^ l^−1^); adequate renal function (glomerular filtration rate (GFR) >40 ml min^−1^ calculated using Cockcroft formula); and adequate liver function (serum bilirubin within normal limits, AST, ALT, ALP <1.5 times upper limit of normal (ULN), or up to 2.5 times ULN in patients with liver metastases and ALP allowed to 2.5 times for bone metastases). Women of child-bearing potential required a negative pregnancy test prior to entry and both men and women were required to use an adequate contraceptive method, to be continued for 3 months after the study.

Patients were given a trial information leaflet and gave written informed consent before recruitment into the trial.

### Response assessment

All patients underwent radiological assessment after completing three cycles of treatment. Patients responding to the treatment or showing stable disease continued on treatment to a maximum of six cycles following which a post-treatment CT scan was performed. Subsequent CT scans were carried out at 3 monthly intervals.

### Statistical considerations

All patients receiving treatment were included in the toxicity assessment. Survival analysis was performed on an intention-to-treat basis. Data were frozen on 1st December 2003. Response and toxicity data were analysed using simple descriptive statistics. Survival curves were calculated according to the method of Kaplan and Meier.

## RESULTS

### Patient characteristics

A total of 32 patients entered the trial from March 2001 to December 2002. Patient demographics are summarised in Table 3

**Table 3:** Patient characteristics

Patient number	32
	
*Tumour grade*	
II	1
III	31
	
*Stage*	
T1	2
T2	3
T3A	2
T3B	7
T4	18
N0	9
N1	23
M0	13
M1	19

. The median age was 66 (range 41–79) years, 22 male, 10 female. A total of 19 patients had visceral metastasis and 22 had lymph node metastasis. Seven patients previously had cystectomy. Eight patients had previous radiotherapy, of which three had concurrent chemoradiation. Three had previous platinum-based chemotherapy. A total of 13 patients had a calculated GFR between 40 and 50 and six between 50 and 60 ml min^−1^.

Detailed analysis was carried out at the time of censor date (1st December 2003). In total, 23 patients received treatment at dose level 1 and nine patients at dose level 2. Once the DLT was achieved, the trial continued to recruit patients in the phase II part of the study at the dose level immediately below the maximum-tolerated dose. In all, 151 cycles of gemcitabine and cisplatin chemotherapy in a 3 weekly schedule were delivered. Toxicity assessment is based on 151 cycles of chemotherapy delivered to these 32 patients.

### Toxicity

At dose level 1, there was one episode of grade IV gastrointestinal perforation, which was thought to be unrelated to trial medication and so the patient was replaced in the cohort. No DLT was observed at dose level 1 and therefore subsequent patients were recruited to dose level 2.

At dose level 2, nine patients were treated. Toxicity is summarised in Table 4

**Table 4:** Summary of toxicities

**Toxicity**		**CTC grade**	**Dose level 1 (23 patients)**	**Dose level 2 (nine patients)**
Haematological	Thrombocytopenia	3	10	
		4	2	3 (DLT)
	Neutropenia	3	9	
		4		2 (DLT)
	Anaemia	3	4	
		4		
Gastrointestinal	Perforation	4	1	
	Haemorrhage	4	1	
	Other	4	1	
				
Nausea		2	4	
		3	2	
Neurological		2	1	

. Three patients experienced grade IV thrombocytopenia, thus defining the DLT. Two of nine patients also developed grade IV neutropenia.

The phase II portion of the study then proceeded using dose level 1. In total, 23 patients have been treated at dose level 1, four in phase I and 19 in phase II. Grade III/IV thrombocytopenia occurred in 12 of 23 patients, occurring on day 15 but with no clinical sequelae.

In terms of nonhaematological toxicity, there were three early deaths. One patient developed gastrointestinal perforation while taking steroids as an antiemetic. One patient developed gastrointestinal bleeding while on warfarin for deep venous thrombosis. A further patient developed abdominal pain while on chemotherapy. This prompted laparatomy, at which no intra-abdominal pathology was evident. However, the patient subsequently died.

No diarrhoea, ototoxicity, nephrotoxicity, or pulmonary toxicity has been observed. One patient developed grade II peripheral neuropathy 10 weeks after completing chemotherapy treatment.

### Response to therapy

Efficacy analysis has been performed on an intention-to-treat basis. Four patients achieved a CR (four of 32, 12.5%) and 17 patients had a PR (17 of 32, 53%) to treatment. Thus, an overall response rate of 65.5% (21 of 32) was seen. Six patients had stable disease and one patient had disease progression. Four of the 32 patients did not undergo radiological response assessment; three in view of early death, and one patient declined any further treatment after first week of chemotherapy treatment. Response rate for patients who underwent radiological reassessment was 75% (21 of 28). Response assessment is summarised in Tables 5

**Table 5:** Response assessment

**Response**	**Patient number (%)**
CR	4 (12.5%)
PR	17 (53%)
SD	6 (19%)
PD	1 (3%)
NA	4 (12.5%)

CR=complete response; PR=partial response; SD=stable disease; PD=progressive disease; NA=not assessable.

and 6

**Table 6:** Response assessment by stage

**Stage**	**Patient number**	**Objective response: CR+PR (number (%))**
T_4_ N_0_ M_0_	2	1
T_1–4_ N_1_ M_0_	11	9 (82%)
T_1–4_ N_0–1_ M_1_	19	11 (58%)

CR=complete response; PR=partial response.

**Figure 1:**
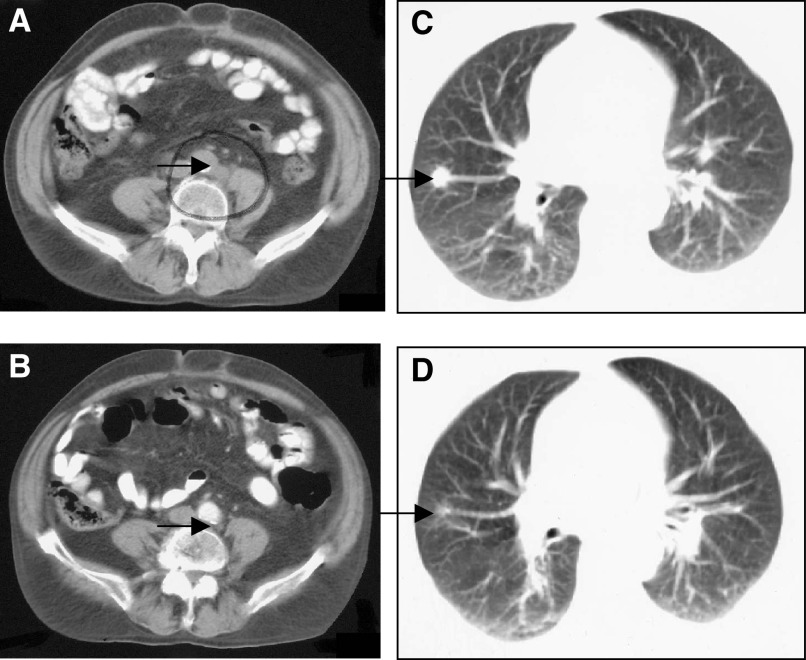
Radiological response assessment. (**A**) Pretreatment-enhanced axial CT scan through mid-abdomen demonstrates enlarged para-aortic nodes between aorta and left psoas muscle (arrow). Post-treatment scan at same level (**B**) demonstrates regression of nodal disease, a tiny focus of residual density is apparent (arrow). (**C**) Pretreatment axial CT through thorax demonstrates a measurable soft tissue nodule in the right middle lobe on lung window settings (arrow); post-treatment (**D**) the nodule has reduced in size, a small ill-defined density is still apparent (arrow).

. Examples of radiological responses are shown in Figure 1

.

### Survival

**Figure 2:**
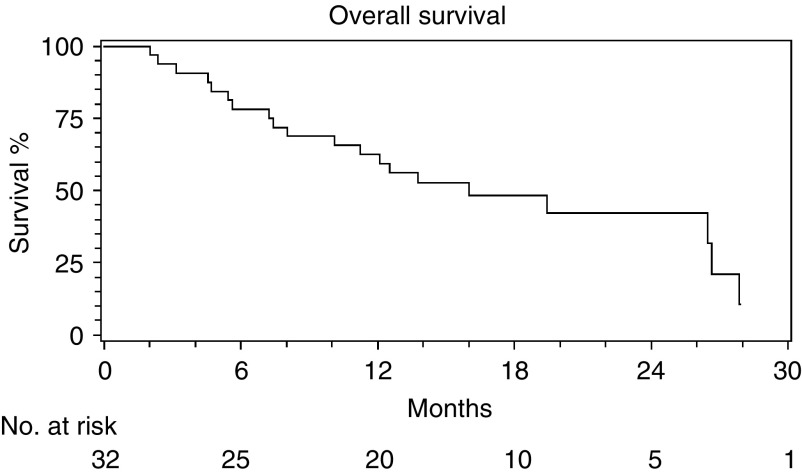
Survival.

At the time of censor, 20 of the 32 patients had died. The median follow-up for the 12 patients remaining alive was 17.2 months (range 13.1–32.4 months). Survival data are summarised in Figure 2

. The median survival for all patients was 16.0 months (10.1–26.6 months) and for the 23 patients treated at dose level 1 was 13.8 months (10.1–yet to be achieved).

### Renal function

**Figure 3:**
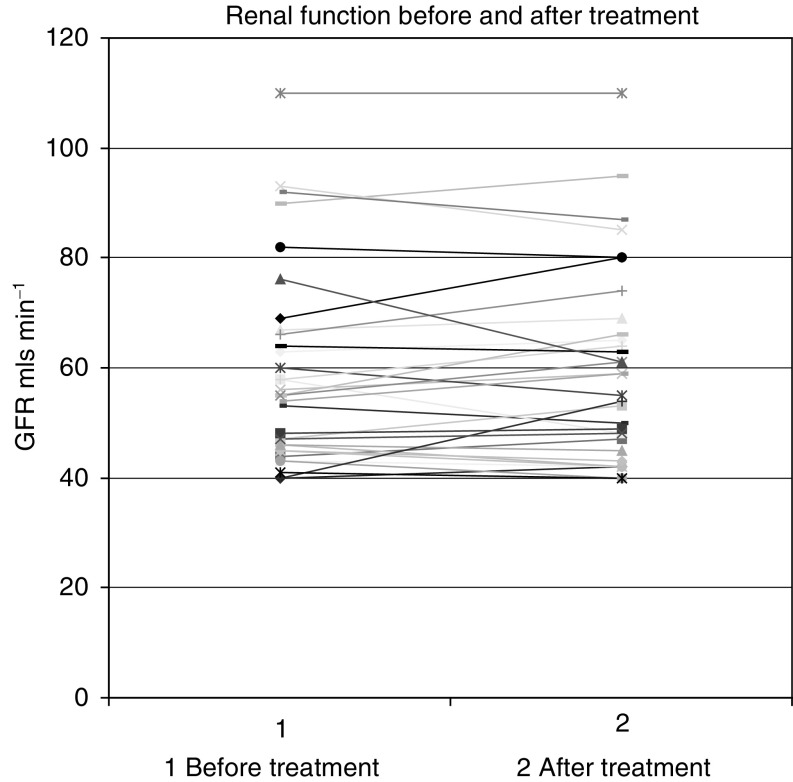
Effect of chemotherapy on renal function.

In only one case chemotherapy was deferred for a week because of renal toxicity. This patient received the chemotherapy a week later as renal function improved following hydration. No clinically significant decline in renal function was noted in any patient during this study (Figure 3

). This is encouraging particularly since patients with a GFR of >40 ml min^−1^ were included in this study. This is much less than the cutoff level GFR of 60 ml min^−1^ employed by many of the reported trials using cisplatin-based combination chemotherapy. Indeed, half of the patients in this study had a GFR <60 ml min^−1^, which may have excluded them from other cisplatin-based trials.

### Dose intensity

This regimen was well tolerated, with most haematological toxicities occurring on day 15, the planned rest day. Thus, we were able to deliver chemotherapy drugs as planned in a majority of cases, the median dose intensity delivered being 92% (range 75–100%). On day 15, platelets were <100 in 61 of 151 cycle of chemotherapy, which would have necessitated dose modification in the standard GC schedule (Von Der Maase *et al*, 2000). Furthermore, on day 15, a neutrophil count of <0.5 and/or a platelet count of <50 was seen in 26 of 151 cycles of treatment, which would have led to dose omission in the 28-day schedule (treatment days 1, 8, and 15). These data advocate the case for a 21-day schedule as a means of increasing the delivered dose intensity of the combination of gemcitabine and cisplatin.

## DISCUSSION

The hope from the early 1980s that metastatic bladder cancer would one day be cured using chemotherapy is yet to be realised. It is important that new combinations and new schedules are evaluated in a clinical trial setting to improve the outcome for patients with this disease. This study using gemcitabine and cisplatin in a 21-day schedule in an outpatient setting illuminates many interesting aspects of chemotherapy treatment. This combination has been used previously but in an in-patient setting and in trials where patient entry criteria of GFR >60 ml min^−1^ would have excluded half of the patients treated in our trial.

In the series presented here 60% of patients had visceral metastases, a known poor prognostic indicator. In addition, eight patients had relapsed after radical radiotherapy, seven had relapsed after cystectomy and three had disease progression after previous cisplatin-based chemotherapy. Taking into account these unfavourable characteristics, a response rate of 65.5% in this group of patients is encouraging and is comparable with many studies using standard chemotherapy treatments and reports of other new treatment schedules. We report a CR rate of 12.5%, which is very similar to that reported in the published literature. The median survival of 16 months in this group of patients is, again, encouraging. These results, along with a favourable toxicity profile, mandate further investigation of this schedule in large, prospective randomised studies.

Although not formally assessed in this study, it is likely that quality of life is enhanced by delivering chemotherapy in an outpatient setting compared to in-patient treatment. Outpatient drug delivery also reduces demand for in-patient resources, thus making it a very attractive and cost-effective mode of chemotherapy administration.

The dose intensity achieved with this schedule questions the use of a 28-day schedule and, indeed, further studies of 21-day schedule are warranted. Optimised treatment with increased dose delivered and fewer dose delays and dose modifications due to toxicity may well have an impact on disease free survival and overall survival. Phase III trials addressing this question are required. Such trials will also be able to compare directly the outpatient chemotherapy regimen used in this trial with in-patient chemotherapy protocols so that quality of life studies can also be addressed.

In summary, this study, using a fractionated cisplatin regimen demonstrates that dose delivered can be improved by using day 15 as a rest day from chemotherapy and shortening the cycle length. It also proves that this regimen can be delivered safely in an outpatient setting in a group of patients with GFR as low as 40 ml min^−1^. These findings provide a forward step in broadening the population group who may benefit subjectively and objectively from the use of palliative chemotherapy. This schedule is already in use in pilot studies in other regions of UK.
